# Complications related to short peripheral intravenous catheters in patients with acute stroke: a prospective, observational, single-cohort study

**DOI:** 10.1007/s11739-024-03651-2

**Published:** 2024-05-28

**Authors:** Daniele Privitera, Annalisa Geraneo, Greta Li Veli, Giorgio Parravicini, Annamaria Mazzone, Michela Rossini, Marianna Sanfilippo, Alessandro Gubertini, Chiara Airoldi, Nicolò Capsoni, Erica Busca, Erika Bassi, Thomas Langer, Alberto Dal Molin

**Affiliations:** 1 Department of Biomedicine and Prevention, University of Rome Tor Vergata Rome, Rome, Italy; 2 Department of Emergency Medicine, ASST Grande Ospedale Metropolitano Niguarda, Milan, Italy; 3 Department of Translational Medicine, Università del Piemonte Orientale, Novara, Italy; 4 Health Professions’ Direction, Maggiore Della Carità Hospital, Novara, Italy; 5 Department of Medicine and Surgery, University of Milan-Bicocca, Monza, Italy; 6 Department of Anesthesia and Intensive Care Medicine, Niguarda Ca’ Granda, Milan, Italy

**Keywords:** Stroke, Emergency department, Vascular catheter, SPC, Device removal, Complications

## Abstract

Patients with acute stroke often require venous access to facilitate diagnostic investigations or intravenous therapy. The primary aim of this study was to describe the rate and type of complications associated with the placement of a short peripheral catheter (SPC) in patients with acute ischemic or hemorrhagic stroke. A prospective, observational, single-cohort study was conducted at Niguarda Hospital, Italy, with enrolment in the Emergency Department. Adult patients with an ischemic or hemorrhagic stroke requiring an SPC were enrolled. Complications, such as infiltration, occlusion, phlebitis and dislodgment, were recorded daily. Descriptive statistics were used, and the incidence rate ratio (IRR) was estimated to assess the difference in complications, considering catheter calibre, dominant side, exit site, limb, and limb mobility, ictus type (ischemic/haemorrhagic), impairment deficit (language, motor, visual) and EA-DIVA score. A total of 269 participants and 755 SPC were analysed. Removal of SPC due to at least one local complication occurred in 451 (60%). Dislodgment was the major cause of SPC removal (31%), followed by infiltration (18%), occlusion (6%), and phlebitis (5%). The SPC calibre (22G), exit-site other than antecubital and forearm, visual deficit and EA-DIVA ≥ 8 were associated with a higher rate of SPC complications: IRR, 1.71 [1.31; 2.31]; 1.27 [1.01; 1.60], 1.38 [1.06; 1.80], 1.30 [1.04; 1.64], respectively. No other differences in complication rates were observed according to the insertion site, i.e. dominant side, left side, plegic/hyposthenic limb, or exit site. This study provides novel insights into the frequency and types of complications associated with SPC in patients with acute stroke. Compared to the literature, a higher dislodgment rate was observed, being the first cause of SPC removal, whereas no differences in the number of infiltrations, occlusions, and phlebitis were recorded.

**Supplementary Information:**

The online version contains supplementary material available at 10.1007/s11739-024-03651-2.

## Background

In Europe, stroke affects about 1.1 million inhabitants per year, leading to approximately 440,000 deaths [1]. The emergency department is the entry point to the healthcare system for most stroke victims [2]. Patients with stroke usually require the placement of a venous access, both for diagnostic investigations and intravenous therapy [2, 3]. As confirmed in the recent guidelines of the Infusion Nursing Society (INS) 2024, in the presence of a plegic or paretic arm (e.g., traumatic injury, cerebrovascular accident), the affected extremity should not ideally be the site of venous access, as alteration in normal blood flow and decreased sensation could be present [4]. Furthermore, several studies showed a significant loss of global muscle mass in patients with acute ischaemic stroke over a 2-week period, and the loss was more pronounced in the upper limbs [5, 6]. Finally, these patients may require an ultrasound assessment to determine the safest location and size of the vascular access device [4]. Regarding complications occurrence, a recent meta-analysis reported a 36% rate of phlebitis and a 24% rate of infiltration [7], whereas occlusions or dislodgments were observed less frequently [8–11]. The failure of SPC can cause pain, anxiety, therapy interruption, infection-related morbidity and mortality, and requires additional procedures for catheter replacement, leading to significant increases in healthcare costs and workloads [12]. To date, there are no studies available that describe which complications and how often they occur in stroke patients. The observation of the most common complications related to the presence of an SPC in stroke patients may lead to a better understanding of the phenomenon, improving care and management of vascular access in this particular population. Based on these premises, the primary aim of this study was to describe the rate and type of complications related to SPC placement in patients affected by ischemic or hemorrhagic stroke. The secondary objective was to measure the risk of developing the complications that occurred, taking into account the factors considered (calibre, dominant side, exit site, limb mobility and side).

## Methods

### Study design

A single-center, prospective, observational, single-cohort study was conducted. The study was approved by the local Ethical Committee (CEMIA3 no. 276-20042022), and informed consent was obtained from all the participants.

### Setting

The study was conducted at ASST Grande Ospedale Metropolitano Niguarda, Milan, Italy, and the enrolment was conducted at the Emergency Department between May 2022 and January 2023. As a multispecialty hospital, Niguarda hosts all the disciplines for adults and children (e.g., Trauma Center with Burn Intensive Care Unit, a Tissue Bank, an Anti-Poisoning Center, and a Stroke Unit). Patients were recruited in the Emergency Department; then, after being transferred to the designated hospital unit, patients were observed and assessed every 24 h until discharge, death or placement of a medium/long-term venous catheter (central or peripheral). Data on each SPC were collected at baseline (t0), i.e., during the insertion, and until removal.

### Participants

The study population consisted entirely of patients characterized by sudden, non-convulsive loss of neurological function due to cerebral ischemia or intracranial hemorrhage and needing peripheral venous access for fluid, drugs or contrast infusion, blood drawing, or transfusions.

*Inclusion criteria* were newly diagnosed ischemic or hemorrhagic stroke [13]; age ≥ 18 years; need for SPC.

*Exclusion criteria* were age < 18 years, patients with Transient Ischemic Attack, defined as brief episodes of neurological dysfunction resulting from focal cerebral ischemia not associated with permanent cerebral infarction [14]; the presence of central venous access device (CVAD) already in place or indication to CVAD insertion (infusion of vesicant drugs or prolonged infusion (> 30 min) of peripherally incompatible solutions; repeated daily blood sampling; hemodialysis; need for hemodynamic monitoring; need for long-term intravenous access (> 3–4 months)[15].

### SPC positioning technique and management

If the identified vein was visible and palpable, a standard insertion technique was used (blind approach). Otherwise, in participants with known difficult vascular access according to Enhanced Adult DIVA (EA-DIVA) [16], the short-axis/out-of-plane ultrasound approach was used [17]. Ultrasound was performed using a portable ultrasound machine (MyLab Alpha, Esaote Spa, Florence, Italy). The examination was carried out using a linear transducer (5–14 MHz). Braun Vasofix^®^ Safety B catheters, 22, 20, or 18 Gauge (G), polyurethane, with 2.5, 3.3, and 4.5 cm lengths, respectively, were used. The calibre of the catheter was decided by the nurse, depending on the intended use (infusion of crystalloids, drugs, blood or contrast medium). Staff dedicated to SPC insertion were only nurses.

The SPC insertion, site selection and management were made according to the recommendations of the INS guidelines and ERPIUP consensus [4, 15]: (i) where possible, avoid venipuncture on an extremity with paralysis or hemiparesis; (ii) possibly choose forearm vessels to prolong the dwell time, decrease pain, and prevent accidental removal and occlusions, and (iii) possibly avoid SPC insertion in areas of flexion; (iv) each SPC was inserted after proper hand hygiene, skin cleansing with a proper antiseptic with 2% chlorhexidine, clean gloves and aseptic technique. After each use, catheter flushing was performed with preservative-free 0.9% sodium chloride [4, 12]. 3 M™ Tegaderm IV Advanced, a borderer transparent dressing, was applied to protect the insertion site and secure the SPC. In addition, to minimise the risk of infection, 2% chlorhexidine in alcohol was used to clean the exit site when a dressing change was required [4, 12]

### Outcomes

The following outcomes were measured: (i) *Infiltration*, defined as the permeation of intravascular fluid into the interstitial compartment, causing swelling of the tissue around the catheter site [7]. The evaluation was based on the clinician’s judgment. The event could occur at any time during the hospital length of stay. (ii) *Phlebitis*, defined when pain, erythema of the skin, swelling and palpable thrombosis of the encysted vein was present [7]. This was assessed using the Phlebitis Scale [18] (range 0–4) with a score ≥ 1; (iii) *Occlusion,* defined as the inability and/or impossibility to infuse fluids through the catheter due to an obstruction [7]; (iv) *Dislodgment* of the SPC, defined as accidental removal that resulted in the loss of function of the catheter [8].

### Data sources/measurement

Patient characteristics, admission unit, SPC insertion, and study outcomes were collected daily during hospitalization. Medical records were accessed to retrieve the admission unit and the following patient’s characteristics: identification code, date of birth, gender, triage level, type of stroke, and kind of deficit (motor, visual, and language). About SPC insertion, the following information was collected: number of venipunctures for each SPC positioned, number of SPCs inserted, date and time of SPC placement, implant site (dominant/non-dominant arm; left or right; plegic arm, hyposthenic, or preserved mobility), catheter size, use of ultrasound/blind approach, SPC use (blood drawing, intravenous therapy, contrast medium, and transfusions), presence of blood return, time and cause SPC removal, EA-DIVA score. EA-DIVA Score values range from 0 to 12, and a cutoff > 8 identifies a patient with difficult intravascular access [16]. Intra-procedural pain, defined as an unpleasant sensory and emotional experience associated with or resembling that associated with actual or potential tissue damage, was measured using a validated numerical rating scale (NRS) [19, 20]. Data were collected and managed using the REDCap (Research Electronic Data Capture; Vanderbilt University, TN) tools hosted at the University of Eastern Piedmont.

### Sample size

Among the possible outcomes of interest, infiltration was considered for sample size calculation. Based on the available literature indicating an infiltration rate of 24% [7], at least 278 patients were required, with a 95% confidence interval and a width of 10%.

### Statistical analysis

Continuous variables are presented as mean with standard deviation (SD) or median and interquartile range (IQR), as appropriate. Categorical variables are expressed as frequencies (percentages).

The hours/days catheters were obtained for each SPC positioned, and the rate among the number of infiltrations and hospitalisation time per person was calculated. The estimates were reported with 95% confidence intervals [95% CI]. Proportions and rates were also calculated for each removal reason. Finally, Poisson models using the time as offset were considered, and incidence rate ratios (IRR) were estimated to assess the difference of adverse events considering calibre catheter, exit-site, kind of deficit, dominant side, limb mobility, ictus type (ischemic/haemorrhagic), impairment deficit (language, motor, visual) and EA-DIVA score. Moreover, Kaplan–Meier curves, both with 95% confidence intervals, were reported for the main outcomes. All the analyses were conducted using the software SAS 9.4 and STATA 15; significant thresholds were set to 0.05 (two-tailed).

## Results

### Characteristics of participants

Two hundred and eighty participants were recruited, for a total of 831 SPC. Of these, 11 participants (4%) and 76 SPCs (9%) were subsequently excluded as they were exposed to non-peripheral compatible therapy during hospitalisation. The sample was prevalently composed of males (*n* = 153, 57%), and the mean age was 74 ± 12, ranging between 42 and 102 years. Two hundred and thirty participants (86%) had an ischemic stroke, and the more prevalent deficit was related to movement (*n* = 218, 81%), followed by language (*n* = 146, 54%) and visual impairment (*n* = 27, 10%). Particularly, 109 (41%) had only a movement deficit, 41 (15%) had only a language deficit, and 10 (4%) only visual ones; movement and language deficits were observed for 92 (34%) of the sample, five (2%) had movement and visual deficit while 1 patient had language and visual deficit; for 12 (4%) subjects’ deficits were observed for the three conditions. Patients were mostly admitted to the Stroke Unit (*n* = 200, 74%). In addition, only 50 participants (19%) presented an EA-DIVA score ≥ 8. More details are reported in Table 1.

**Table 1:** Number, proportion, and incidence rate (× 1000 device-hours and × 1000 device-days) of SPC removal shown by major causes

Causes	N^o^	Proportion	Rate (× 1000 device-hours)	Rate (× 1000 device-days)
		% [95% CI]		
Dislodgement	236	31 [27.95; 34.56]	5 [4.11; 5.30]	112 [95.57; 127.22]
Infiltration	138	18 [15.58; 21.22]	3 [2.31; 3.22]	65 [55.42; 77.37]
Occlusion	47	6 [4.50; 7.95]	1 [0.70; 1.24]	22 [16.76; 29.638]
Phlebitis	39	5 [3.59; 6.99]	1 [0.56; 1.06]	19 [13.52; 25.33]
Other	12	2 [0.70; 2.48]	0 [0.13; 0.42]	6 [3.23; 10.03]
SPC removal by at least one complication	451	60 [56.24; 63.23]	9 [8.13; 9.78]	214 [195.13; 234.69]

### SPC insertion

Among the 269 patients included in the study, a total of 755 catheters were inserted. The median number of SPCs per participant was 2 [IQR 1–4], ranging from 1 to 10. Particularly, 78 (29%) participants had only one, 83 (31%) had two, while the remaining participants had three or more SPCs. The average SPC dwelling time was approximately 67 h per catheter, with a median of 60 h [IQR 36–108].

The dominant side was the most selected (*n* = 395, 52%). The SPC was placed mainly in the forearm and antecubital vein, accounting for 37% each. Among the 50 patients with an EA-DIVA score ≥ 8, only 15 participants (2%) required SPC insertion with ultrasound. Furthermore, the limb with preserved mobility was preferred, and only in 13% of cases was the SPC placed in the plegic or hypostenic limb, respectively. The SPCs were mainly placed in the Emergency Department (*n* = 342, 45%), followed by Stroke Unit and Neurology unit (*n* = 267, 35% and *n* = 130, 17%, respectively). The main use of the SPC was for infusion of therapy (92%), followed by blood drawing (42%) and median contrast infusion (32%). Further descriptive statistics of the SPCs positioned are reported in Table 2.

**Table 2:** Dislodgment stratified for calibre, dominant side, exit site, limb mobility, side, kind of ictus and deficit, and EA-DIVA score

	Dislodgment		
	N^o^ events	Rate [95% CI]	IRR [95% CI]
Calibre			
18G	88	94 [76.29; 115.86]	1
20G	115	116 [97.00; 139.81]	1.24 [0.94; 1.63]
22G	33	179 [127.50; 252.27]	**1.91 [1.28; 2.85]**
Dominant side			
No	108	106 [87.64; 127.80]	1
Yes	128	118 [99.02; 140.03]	1.11 [0.86; 1.44]
Exit site			
Forearm	90	113 [92.31; 139.54]	1
Antecubital vein	79	97 [77.51; 120.48]	0.85 [0.63; 1.15]
Others	67	135 [106.10; 171.28]	1.19 [0.87; 1.63]
Limb mobility			
Preserved mobility	173	113 [96.94; 130.60]	1
Plegic/paretic	32	105 [74.44; 148.85]	0.94 [0.64; 1.36]
Hyposthenia	31	117 [81.96; 165.71]	1.04 [0.71; 1.52]
Limb			
Right	130	124 [104.45; 147.31]	1
Left	106	100 [82.70; 121.03]	0.81 [0.63; 1.04]
Type of ictus			
Ischemic	180	105 [90.90; 121.75]	1
Haemorrhagic	56	141 [108.69; 183.52]	1.34 [0.99; 1.81]
Language deficit			
No	93	99 [80.53; 120.91]	1
Yes	143	123 [104.19; 144.61]	1.24 [0.96; 1.62]
Motor deficit			
No	35	112 [80.16; 155.49]	1
Yes	201	112 [97.57; 128.65]	1.00 [0.70; 1.44]
Visual deficit			
No	208	111 [96.48; 126.61]	1
Yes	28	124 [85.73; 179.83]	1.12 [0.76; 1.67]
EA-DIVA score			
< 8	187	106 [91.98; 122.52]	1
≥ 8	49	142 [107.03; 187.38]	1.33 [0.98; 1.83]

### Outcomes of the study

**Fig. 1:**
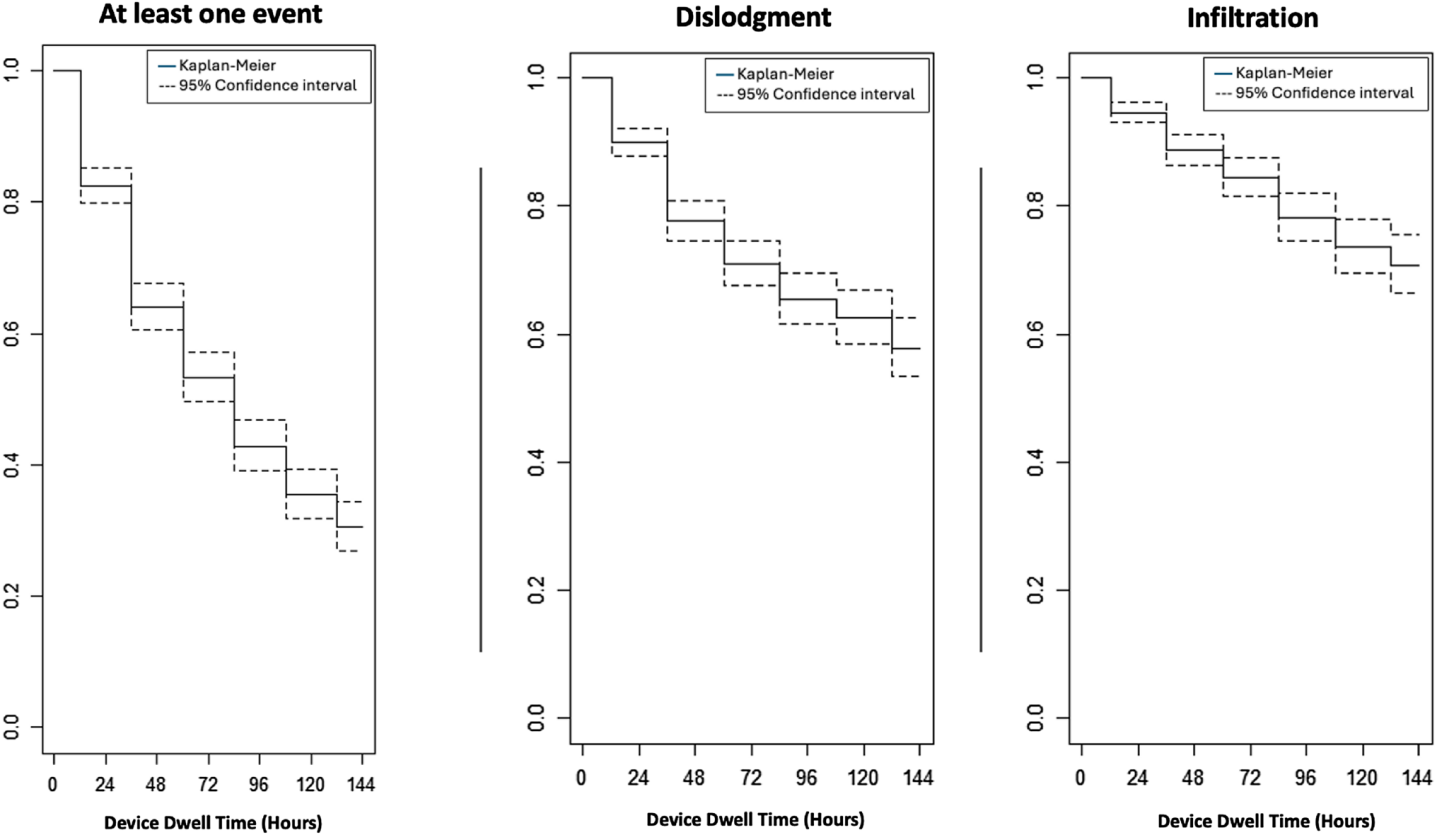
From left to right: Kaplan–Meier curve of (i) at least one event at removal, (ii) dislodgment, and (iii) infiltration at removal

Overall, 451 SPCs were removed due to at least one local complication (60% and 214 per 1000 device). The major cause of SPC removal was the dislodgment observed 236 times (31%), followed by infiltration observed in 138 cases (18%) (Table 1). Less frequent were occlusion and phlebitis (6% and 5%, respectively). Considering that the total time of observation was of 2107 days, the dislodgement incidence rate was 112 per 1000 device-days, followed by infiltration (rate 65), occlusion (rate 22) and phlebitis (rate 19). Additional analyses were performed, stratifying major removal causes by SPC calibre, dominant side, exit site, limb mobility and side, ictus type (ischemic/haemorrhagic), impairment deficit (language, motor, visual) and EA-DIVA score (Tables 2, 3 and Table 3). Kaplan–Meier survival estimates for at least one event, dislodgement and infiltration, over the first 144 h of catheter dwell are shown in Fig. 1.

**Table 3:** Infiltration stratified for calibre, dominant side, exit site, limb mobility, side, kind of ictus and deficit, and EA-DIVA score

	Infliltration		
	N^o^ events	Rate [95% CI]	IRR [95% CI]
Calibre			
18G	60	64 [49.77; 82.56]	1
20G	62	63 [48.95; 80.53]	0.98 [0.69; 1.40]
22G	16	87 [53.27; 141.94]	1.36 [0.78; 2.35]
Dominant side			
No	67	66 [51.67; 83.41]	1
Yes	71	65 [51.76; 82.42]	0.99 [0.71; 1.39]
Exit site			
Forearm	57	72 [55.44; 93.19]	1
Antecubital vein	52	64 [48.47; 83.47]	0.88 [0.61; 1.29]
Others	29	58 [40.55; 83.97]	0.81 [0.52; 1.27]
Limb mobility			
Preserved mobility	107	70 [57.58; 84.11]	1
Plegic/paretic	13	43 [24.83; 73.64]	0.61 [0.35; 1.09]
Hyposthenia	18	68 [42.63; 107.40]	0.97 [0.59; 1.60]
Limb			
Right	71	68 [53.69; 85.49]	1
Left	67	63 [49.77; 80.35]	0.93 [0.67; 1.30]
Type of ictus			
Ischemic	109	64 [52.80; 76.86]	1
Haemorrhagic	29	73 [50.83; 105.25]	1.15 [0.76; 1.73]
Language deficit			
No	58	62 [47.57; 79.60]	1
Yes	80	69 [55.16; 85.49]	1.12 [0.80; 1.56]
Motor deficit			
No	25	80 [53.88; 118.02]	1
Yes	113	63 [52.38; 75.74]	0.79 [0.51; 1.22]
Visual deficit			
No	107	57 [47.04; 68.72]	1
Yes	31	137 [96.68; 195.48]	**2.42 [1.62; 3.61]**
EA-DIVA score			
< 8	106	60 [49.74; 72.79]	1
≥ 8	32	92 [65.40; 130.78]	**1.54 [1.04; 2.28]**

Of 342 (45%) SPCs inserted in ED, 199 (58%) were removed due to local complications, compared to 252/413 (61%) in other units. The incidence rate of complications in ED was 192.62 [95% CI 166.77; 220.18] per 1000 device-days compared to 235.73 [95% CI 208.35; 266.71] observed in other units. A statistical difference (*p* < 0.0001) was observed in terms of catheter calibre between ED and other units: 18G catheters were mainly used in ED (75% vs 14%), while 20G catheters were prevalently used in other units (66% vs 24%).

## Discussion

The present study aimed to describe types and rates of complications related to SPC inserted in patients diagnosed with acute ischemic or hemorrhagic stroke admitted to the ED. In our study, subjects with ischemic stroke accounted for most of the patients observed, while the most frequent deficit was motor impairment, followed by language and visual impairment. The population characteristics observed in this study were similar to those already described in literature [21].

Throughout the study, 755 SPCs were inserted, and 60% of them were removed due to at least one local complication. The average SPCs dwell time was 67 h (median 60 h), lower than the results reported in a recent systematic review (3.5 days) [22]. Most of the participants did not show any distinguishable features that would classify them as DIVA patients. This might explain why ultrasound was necessary for only 2% of the sample for the placement of an SPC.

The most common complication in the observed population was dislodgment (31%), with values significantly higher than those described in the literature, ranging from 2 to 10% [22–24]. One possible cause could be the presence of moderate to severe motor/sensory deficits, and/or cognitive deficits, and/or post-stroke rehabilitation care [25], which could lead to accidental catheter removal. Indeed, the more prevalent deficit among participants was related to movement (81%). Interestingly, these results are to be correlated with the use of a bordered transparent dressing: simple and reliable securement and should be the first choice when the SPC must stay in place for several days, as suggested by ERPIUP consensus [15]. In addition, as suggested in a recent study by Bahl et al., we should consider the use of cyanoacrylate glue as a strategy to reduce dislodgement in patients at higher risk of SPC dislodgement [26]

When stratifying the dislodgment rate by the calibre of the catheter, dominant side, exit site, limb mobility, side, and kind of deficit and ictus, the only statistically significant association observed was with the SPC calibre. Dislodgment was higher in participants that had 22G SPCs inserted, with an IRR of 1.91 (95% CI 1.28; 2.85) compared to larger calibres. The shorter length of the 22G SPCs (2.2 cm) when compared to the 20G or 18G catheters (3.3 cm and 4.5 cm, respectively) could explain these findings [4].

Infiltration was the second most common cause of SPC removal (18%), a lower rate than in a Cochrane review, where it occurred in 24% of cases [7]. Typically, previous studies conducted on other patients have shown that the primary complications encountered are phlebitis and infiltrations, with dislodgment and occlusions being secondary concerns [7, 10, 23]. When the infiltration rate was stratified according to risk factors, a statistically significant association was observed between a visual impairment deficit and the EA-DIVA score ≥ 8. In the first case, visual impairment after stroke due to difficulties in navigating/orientating in the environment [27] and altered cognitive status is associated with an increased risk of infiltration [4]. Even in the second case, patients with peripheral vein access difficulties have an increased risk of extravasation and infiltration [4]. Previous study indicates that SPC inserted with difficulty have a higher number of catheter-related adverse events, mainly infiltration, phlebitis and occlusion [28].

However, in our study, we observed less frequent occurrences of occlusion and phlebitis, accounting for 6% and 5%, respectively. One possible explanation for this finding is that in over 95% of cases, the SPC was used for delivering non-phlebitogenic medications, making it suitable for peripheral administration. In addition, the use of all indications recommended by the INS guidelines and ERPIUP consensus has certainly contributed to a reduction in SPC-related complications [4, 15]

The rate of SPCs removal by at least one complication was relatively high (60%), exceeding the findings of a randomised trial where SPC failure ranged between 38 and 43% [12]. The SPCs removal also showed a statistically significant association with 22G calibre, exit site different from that of the forearm or antecubital vein, presence of visual deficit and EA-DIVA ≥ 8. In this study, only 11% of the inserted SPCs had the smallest calibre and were mostly used in patients who only required drug therapy or blood sampling. According to the literature, the larger calibre was primarily placed when a computerised tomography scan with contrast medium was performed [29]. No other statistically significant associations were found when the SPC removal rate was stratified by dominant side, limb mobility, side, type of ictus and presence of language or motor deficits.

Regarding the side of insertion, SPCs were placed with a similar distribution between the dominant and non-dominant limb, in contrast to other studies that recommended catheter insertion in the non-dominant limb for increasing catheter dwell time [8, 30]. This is likely due to the criteria adopted for selecting the insertion side, which favored the limb with preserved mobility after a stroke.

The most chosen sites to insert the SPCs were the antecubital vein (37%) and forearm (37%), as reported by Marsh et al. [8] Despite the literature does not recommend catheter insertion in areas of flexion like the antecubital vein [4, 15], the findings of this study demonstrate no statistically significant association between any type of exit site and dislodgement, infiltration, or SPC removal by at least one of the observed local complications.

A further novelty of this study was to describe the complications associated with SPC insertion in the limb with impaired mobility (plegia or hyposthenia). As said before, the majority of the SPCs were placed in the limb with preserved mobility (74%) according to the recommended practice [4]. Although no statistically significant difference was found, infiltration, dislodgement and SPC removal for at least one complication occurred less frequently if the SPC was placed in the plegic limb, compared to the hyposthenic limb or limb with preserved mobility. These findings appear to contradict the available literature, which advises against the placement of vascular access in limbs with paralysis or plegia [4]. However, the results of this study may be explained by the fact that the loss of muscle tone does not occur in the first few days after the acute event but in the first 2 weeks [5]. Currently, there is a lack of studies that establish whether SPC insertion in the limb with preserved mobility is safer compared to the plegic limb. In order to better understand this point, further studies with a comparison group and adequate sample size are needed.

Future research is needed to confirm the data found in this study, to explore additional factors that may influence complication rates and to develop targeted interventions for optimizing SPC dwelling time in this patient population. It would be worthwhile to assess whether an enhanced nursing monitoring of SPCs placed in a plegic or hypostenic limb could lead to further reduction in SPC complications. Understanding this is particularly important because it could allow for the utilization of the plegic or hypostenic limb and, meanwhile, prevent potential harm associated with decreased sensitivity, which in these patients may hinder the identification of pain associated with occurring complications.

### Strengths and limitations

To the best of our knowledge, this is the first study describing the rate and nature of complications related to SPC in patients with ischemic or hemorrhagic stroke. The main strength of this study was conducting a daily follow-up of participants, which allowed for monitoring of SPC-related complications throughout the patient’s entire hospital stay.

However, this study has several limitations: the generalizability of our results is undoubtedly limited because the study was conducted in a single centre. In addition, risk factors such as delirium, previous dementia or stroke size were not analysed. Furthermore, the nurses on the units involved in the study were aware that stroke patients would be observed by a group of external nurses. This may have unknowingly changed the management of SPC. In addition, we did not consider using long peripheral catheters (LPCs) because this type of catheter was unavailable in our hospital at the time of the study. Perhaps the use of LPCs would have reduced complications by avoiding the placement of numerous SPCs, as described in the literature [31]. A vascular access team was only established shortly after the start of this study. This may explain why some patients needed > 4 SPCs. However, despite several studies highlighting the benefits of hospital-based vascular access teams—such as improving staff expertise through training, optimising catheter monitoring and selection, diversifying the use of vascular access devices, promoting awareness of vascular access policies, facilitating up-to-date vascular access training and supporting systematic complication monitoring [32–35]—it remains uncertain whether specialised vascular access teams outperform the generalist model [36].

## Conclusion

For the first time, this study provides a comprehensive description of the rate and nature of complications related to SPC in patients with ischemic or hemorrhagic stroke, contributing valuable insights into this specific population.

These findings indicate that dislodgment was the primary cause for SPC removal, with significantly higher rates compared to literature data. Interestingly, infiltrations, occlusions, and phlebitis occurred with frequencies comparable to or lower than those reported in existing literature. Moreover, this study revealed that the presence of preserved or altered limb mobility, as well as the placement of the SPC on the dominant or non-dominant limb, did not significantly impact complication rates. Further prospective studies are necessary in this field.

In addition, this study highlights the importance of properly monitoring and managing SPCs regardless of limb mobility status or dominance. It emphasises the need for heightened attention to dislodgment prevention strategies when utilising SPCs in stroke patients.

## Supplementary Information

Below is the link to the electronic supplementary material.

Supplementary file1 (PDF 253 KB)

## Data Availability

All data have been retained within a purpose-built password-protected REDCap database, saved on a secure password-protected shared drive housed at the university of Eastern Piedmont server. All data may be downloaded via a safe encrypted link to which only the listed authors have access.
