# Supplementary material for: Complications related to short peripheral intravenous catheters in patients with acute stroke: a prospective, observational, single-cohort study
Source: Intern Emerg Med. 2024 May 28;19(6):1605–13. doi: 10.1007/s11739-024-03651-2 (PMC11405487; doi:10.1007/s11739-024-03651-2)
Supplement: Supplementary file 1 — Supplementary file1 (PDF 253 KB) [file 11739_2024_3651_MOESM1_ESM.pdf]

Supplementary materials

COMPLICATIONS RELATED TO SHORT PERIPHERAL INTRAVENOUS CATHETERS IN PATIENTS WITH ACUTE STROKE: A PROSPECTIVE, OBSERVATIONAL, SINGLE-COHORT STUDY.

Daniele PRIVITERA, CCRN, MSN, PhD(s)[1], Annalisa GERANEO, RN[2], Greta LI VELI, RN[2], Giorgio PARRAVICINI, CCRN[2], Annamaria MAZZONE, RN[2], Michela ROSSINI, RN[2], Marianna SANFILIPPO, RN, MSN[2], Alessandro GUBERTINI, CCRN[2], Chiara AIROLDI, PhD[3], Nicolò CAPSONI, MD[2], Erica BUSCA, RN, PhD[3,4], Erika BASSI, RN, PhD[3,4], Thomas LANGER, MD[5.6], and Alberto DAL MOLIN, RN, PhD[3,4]

[1] *Department of Biomedicine and Prevention, University of Rome Tor Vergata Rome, Italy.*

[2] *Department of Emergency Medicine, ASST Grande Ospedale Metropolitano Niguarda, Milan, Italy*

[3] *Department of Translational Medicine, Università del Piemonte Orientale, Novara, Italy.*

[4] *Health Professions' Direction, Maggiore della Carità Hospital, Novara, Italy.*

[5] *Department of Medicine and Surgery, University of Milan-Bicocca, Monza, Italy.*

[6] *Department of Anesthesia and Intensive Care Medicine, Niguarda Ca' Granda, Milan, Italy.*

Supplementary table 1. Characteristics of the sample

**Variable**	**N=269**
Gender, male, n (%)	153 (57)
Age, years, mean (SD)	74 (12)
**Triage level**	
White (*ambulatory complaints*), n (%)	1 (0)
Green (*non-urgent*), n (%)	44 (16)
Yellow (*urgent*), n (%)	211 (79)
Red (*emergency*), n (%)	14 (5)
**Type of ictus**	
Ischemic, n (%)	230 (86)
Haemorrhagic, n (%)	39 (14)
**Kind of deficit**	
Language deficit, n (%)	146 (54)
Movement deficit, n (%)	218 (81)
Visual deficit, n (%)	27 (10)
**Number of deficits**	
None, n(%)	9 (3)
One, n (%)	150 (56)
Two, n (%)	98 (36)
Three, n (%)	12 (5)
**EA-DIVA score**	
mean (SD)	4 (3.06)
median [q1-q3]	3 [2; 6]
score ≥ 8, n (%)	50 (19)
**Admission unit**	
Stroke Unit, n (%)	200 (74)
Neurology, n (%)	32 (12)
Neurosurgery, n (%)	3 (1)
General medicine, n (%)	2 (1)
Other, n (%)	32 (12)

Supplementary table 2. Descriptive statistics of the SPC position (t0, Baseline)

**Variable**	(n=755)
**Venipunctures number**	
1, n (%)	533 (73)
2+, n (%)	202 (27)
Median [q1-q3]	1 [1; 2]
**Insertion mode**	
Blind, n (%)	740 (98)
Ultrasound, n (%)	15 (2)
**Exit site**	
Antecubital vein, n (%)	279 (37)
Forearm, n (%)	279 (37)
Wrist, n (%)	82 (11)
Hand, n (%)	80 (11)
Cephalic vein, n (%)	18 (2)
Foot, n (%)	15 (2)
External jugular vein, n (%)	1 (0)
Other, n (%)	1 (0)
**Limb mobility**	
Preserved mobility, n (%)	558 (74)
Plegic/paretic, n (%)	101 (13)
Hyposthenia, n (%)	95 (13)
**Dominant side**	
Dominant, n (%)	395 (52)
Non-dominant, n (%)	360 (48)
**Calibre catheter**	
18 G, n (%)	318 (42)
20 G, n (%)	355 (47)
22 G, n (%)	82 (11)
**Intraprocedural pain**	
Mean (SD)	3 (1.7)
Median [Q1-Q3]	2 [2.00; 4.00]
**SPC use**	
Transfusion, n (%)	6 (1)
IV therapy, n (%)	696 (92)
Median contrast, n (%)	242 (32)
Blood drawing, n (%)	318 (42)

**Catheter insertion unit**	
Emergency department, n (%)	342 (45)
Stroke Unit, n (%)	267 (36)
Neurology, n (%)	130 (17)
Neurosurgery, n (%)	1 (0)
General medicine, n (%)	1 (0)
Other, n (%)	14 (2)

Supplementary table 3. SPC removal stratified for calibre, dominant side, exit site, limb mobility, side, kind of ictus and deficit, and EA-DIVA score.

	At least one event		
	N° events	Rate [95% CI]	IRR [95% CI]
**Calibre**			
18G	176	188 [162.21; 217.97]	1
20G	216	219 [191.43; 249.94]	1.16 [0.95; 1.42]
22G	59	321 [248.44; 413.86]	**1.71 [1.27; 2.29]**
**Dominant side**			
No	207	203 [177.01; 232.44]	1
Yes	244	224 [198.00; 254.48]	1.11 [0.92; 1.33]
**Exit site**			
Forearm	167	211 [180.96; 245.08]	1
Antecubital vein	151	185 [157.48; 216.65]	0.88 [0.70; 1.09]
Others	133	268 [225.78; 317.18]	**1.27 [1.01; 1.60]**
**Limb mobility**			
Preserved mobility	344	217 [195.14; 241.83]	1
Plegic/paretic	60	197 [153.25; 254.20]	0.91 [0.69; 1.20]
Hyposthenia	57	214 [165.29; 277.80]	0.99 [0.74; 1.31]
**Limb**			
Right	243	232 [204.48; 262.94]	1
Left	208	196 [171.37; 224.90]	0.85 [0.70; 1.02]
**Type of ictus**			
Ischemic	351	205 [184.77; 227.77]	1
Haemorrhagic	100	252 [207.32; 306.82]	1.23 [0.98; 1.54]
**Language deficit**			
No	185	196 [169.95; 226.71]	1
Yes	266	228 [202.47; 257.48]	1.16 [0.96; 1.40]
**Motor deficit**			
No	69	220 [173.84; 278.67]	1
Yes	382	213 [192.61; 235.39]	0.97 [0.75; 1.25]
**Visual deficit**			
No	387	206 [186.13; 227.17]	1
Yes	64	284 [222.14; 362.60]	**1.38 [1.06; 1.80]**

**EA-DIVA score**			
<8	359	204 [183.78; 226.01]	1
≥ 8	92	266 [216.75; 326.18]	**1.30 [1.04; 1.64]**
